# Anticancer Potential of *Citrus* Juices and Their Extracts: A Systematic Review of Both Preclinical and Clinical Studies

**DOI:** 10.3389/fphar.2017.00420

**Published:** 2017-06-30

**Authors:** Santa Cirmi, Alessandro Maugeri, Nadia Ferlazzo, Sebastiano Gangemi, Gioacchino Calapai, Udo Schumacher, Michele Navarra

**Affiliations:** ^1^Department of Chemical, Biological, Pharmaceutical and Environmental Sciences, University of MessinaMessina, Italy; ^2^Prof. Antonio Imbesi FoundationMessina, Italy; ^3^Department of Clinical and Experimental Medicine, University of Messina, Messina, Italy and Institute of Applied Sciences and Intelligent Systems, National Research CouncilPozzuoli, Italy; ^4^Department of Biomedical and Dental Sciences and Morphofunctional Imaging, University of MessinaMessina, Italy; ^5^Department of Anatomy and Experimental Morphology, University Medical Center Hamburg-EppendorfHamburg, Germany

**Keywords:** *Citrus* juice, cancer, proliferation, alternative medicine, *in vitro*, *in vivo*, humans, systematic review

## Abstract

**Background:** During the last decades, a huge body of evidence has been accumulated suggesting that *Citrus* fruits and their juices might have a role in preventing many diseases including cancer.

**Objective:** To summarize the numerous evidences on the potential of *Citrus* juices and their extracts as anticancer agents.

**Data sources:** A systematic review of articles written in English using MEDLINE (1946-present), EMBASE (1974-present) and Web of Sciences (1970-present) was performed independently by two reviewers. Search terms included *Citrus, Citrus aurantifolia, Citrus sinensis, Citrus paradisi, Citrus* fruits, *Citrus* fruits extract, cancer, neoplasm, neoplasia, tumor, metastasis, carcinogenesis, proliferation. The last search was performed on March 16th, 2017.

**Study selection:** Study selection and systematic review were carried out in accordance with the Preferred Reporting Items for Systematic Reviews and Meta-Analyses (PRISMA) statement. Prior to the beginning of the review, Authors defined a checklist for inclusion criteria, thus including articles which meet the following: (i) published on peer-reviewed scientific journals; (ii) *Citrus* juice used alone; (iii) extracts derived from *Citrus* juice; (iii) for preclinical studies, an exposure time to *Citrus* juices and their extracts more than 24 h. Reviews, meta-analyses, conference abstracts and book chapters were excluded.

**Data extraction:** Three reviewers independently performed the extraction of articles.

**Data synthesis:** 22 papers met our inclusion criteria and were eligible for inclusion in the final review. According to the kind of study, the selected ones were further divided in preclinical (*n* = 20) and observational (*n* = 2) studies.

**Conclusion:** The studies discussed in this review strongly corroborate the role of *Citrus* juices and their derivatives as potential resource against cancer.

## Introduction

Cancer is a major health problem in the developed world, just after aging-related cardiovascular disease, with 14 million cases worldwide and 8.2 million of deceases just in 2012^1^. This cancer epidemic has a considerable social impact with high socio-economic costs. The fight against cancer is realized by developing innovative drugs and novel therapeutic strategies aimed to cure the disease, as well as to prevent it.

Over the recent decades, we witnessed a return to natural remedies to prevent or treat a large number of degenerative diseases (Paterniti et al., 2014; Cirmi et al., 2016c; Ferlazzo et al., 2016a). Frequently, they are employed when minor health problems occur, however they can even be used as co-adjuvants for synthetic drugs in major health problems. Indeed, natural drugs, supplements and functional foods raised interest in the general population, which more often prefers to use these remedies rather than synthetic ones. This choice is preferred because they are easier to use and exert fewer side effects, thus contributing to increase patients' compliance. Hence, up to 80% of people is using natural remedies worldwide. However, they should not be used empirically, but rather it is greatly important that their effectiveness is evaluated by clinical studies. This thesis led the scientific community to revise the use of some natural medicines employed for decades whom effectiveness has been recently placed in doubt (Tacklind et al., 2012; Micali et al., 2014; Semba et al., 2014).

Another approach to prevent degenerative diseases is following a combination of a healthy life style and diet habits, employing “food as medicines” (Ullah and Khan, 2008). For instance, it has been suggested that high dietary intake of vegetables and fruits (>400 g/day) could prevent at least 20% of all cancer cases (Amin et al., 2009; Gullett et al., 2010). This preventive effect is predominantly exerted by biologically active molecules like flavonoids which are capable of interacting with specific targets that play a role in the generation and progression of cancer (Ravishankar et al., 2013). *Citrus* fruits (CF) are the most eaten fruits in the Mediterranean diet that it is known to reduce the risk of degenerative diseases, including cancer (Giacosa et al., 2013). CF represent one of the most important diet sources of flavonoids (Kozlowska and Szostak-Wegierek, 2014) whose benefits are due to many biological properties, among which the well-known antioxidant activity and the modulation of intracellular key pathways involved in degenerative processes leading to chronic pathologies such as cancer. In particular, dietary flavonoids interfere with carcinogen activation, stimulate carcinogen detoxification, scavenge free radical species, control cell-cycle progression, induce apoptosis, inhibit cell proliferation, oncogene activity, angiogenesis and metastasis as well as inhibit hormones or growth-factor activity (Clere et al., 2011).

The objective of our systematic review is to summarize and critically discuss the numerous evidences on the anticancer properties of *Citrus* juices and their extracts.

## Methods

### Search strategy

Two reviewers (SC and MN) independently performed a bibliographic research in the following electronic databases: MEDLINE (1946-present), EMBASE (1974-present), and Web of Sciences (1970-present). In order to retrieve all relevant papers, no limit was placed on search time frame. The last search was performed on March 16th, 2017.

The databases were searched for relevant studies that included at least one keyword or Medical Subject Heading from each of the following: (i) *Citrus, Citrus aurantifolia, Citrus sinensis, Citrus paradisi, Citrus* fruits, *Citrus* fruits extract; (ii) cancer, neoplasm, neoplasia, tumor, metastasis, carcinogenesis, proliferation. The search was also limited to English language. An example of full electronic search strategy for EMBASE is provided in Table 1. Citations, titles and abstracts were exported into Endnote X5.

**Table 1:** Full electronic search strategy for EMBASE.

**Search strategy for EMBASE**	
**Limit applied: “English”**	
1. *Citrus*	8. tumor
2. *Citrus* fruits	9. metastasis
3. *Citrus* fruits extract	10. carcinogenesis
4. 1 OR 2 OR 3	11. proliferation
5. cancer	12. 5 OR 6 OR 7 OR 8 OR 9 OR 10 OR 11
6. neoplasm	13. 4 AND 12
7. neoplasia	

Study selection and systematic review were performed in accordance with the Preferred Reporting Items for Systematic Reviews and Meta-Analyses (PRISMA) statement (Liberati et al., 2009). Prior to the beginning of the review, Authors defined a checklist for inclusion criteria, thus including articles that meet the following: (i) published on peer-reviewed scientific journals; (ii) *Citrus* juice used alone; (iii) extracts derived from *Citrus* juice; (iii) for preclinical studies, an exposure time to *Citrus* juices and their extracts more than 24 h. Consequently, we excluded: (i) studies performed using preparations derived from other fruits or other parts of the CF (i.e., peel, seeds, whole fruit, etc.); (ii) studies in which the *Citrus* juices were used in combination with other fruits or juices; (iii) studies carried out with isolated molecules extracted from *Citrus* juices. We also excluded reviews, systematic reviews, meta-analyses, letters, personal opinions, conference abstracts and book chapters.

Potentially relevant articles were screened independently by three reviewers (SC, AM, and MN), initially by abstract and then by full text. Two reviewers had previous experience with systematic review (SC and MN). Disagreements among reviewers were resolved by consensus discussions.

## Results

### Study selection

**Figure 1:**
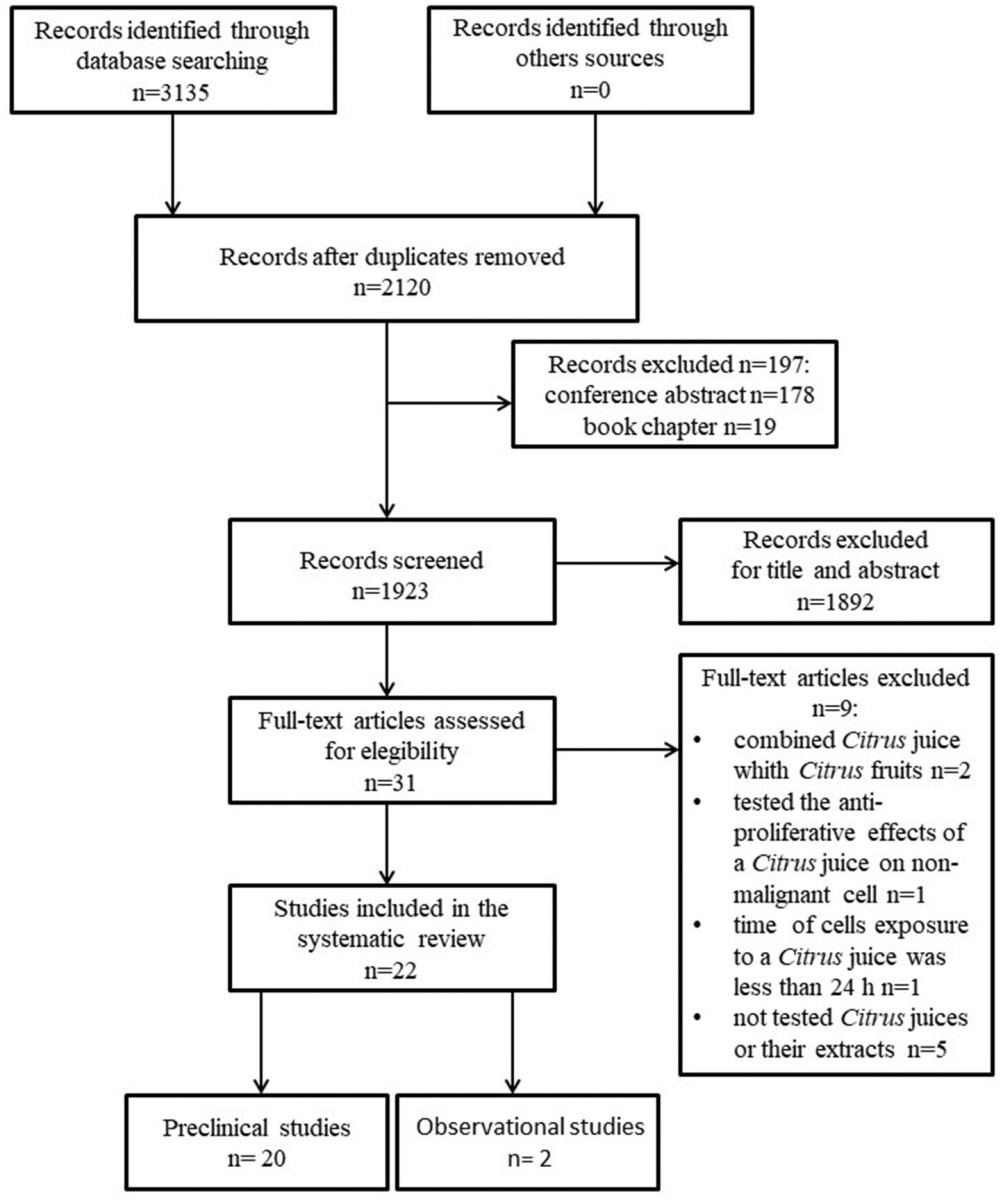
PRISMA flow chart showing the process of literature search and study selection.

The initial electronic databases search resulted in 3,135 articles that were reduced to 2120 when duplicates were discarded. Of these latter, 31 articles were selected for detailed review, with only 22 ones eligible for inclusion in the final review process. Figure 1 shows the flow of papers through the review. According to the kind of study, the selected ones were further divided in preclinical (*n* = 20) and observational clinical studies (*n* = 2). Tables 2–4 summarize the different variables of all studies taken into account. Articles excluded because they were conference abstracts or book chapters were *n* = 197. Records excluded for title and abstract were *n* = 1892. Articles excluded after full text review were *n* = 9: two because combined *Citrus* juice with CF, one because tested the anti-proliferative effects of a *Citrus* juice on non-malignant cells, one because cells exposure time to a *Citrus* juice was less than 24 h and five because did not test *Citrus* juices or their extracts.

**Table 2:** Principal characteristics of the *in vitro* studies testing the anticancer effect of *Citrus* juices or their extracts.

**Study**	***Citrus* juices and extracts**	**Cells**	**Concentration of treatment**	**Time of exposure**	**Inhibition of proliferation**	**Method using to evaluate the growth rate**
Kawaii et al., 1999	34 *Citrus* juices	A549 B16 CCRF-HSB-2 TGBC 11TKB	100 μg/ml	72 h	All the tested juices showed antiproliferative activity, with the most exerted by *C. nobilis*. A549 cell: 48.1% ± 3.2 B16 cell: 50.6%± 3.1 CCRF-HSB-2 cell: 87.8%±2.8 TGBC11TKB cell: 65.3%±1.8	Alamar Blue assay
Fernandez-Bedmar et al., 2011	*C. limon* and *C. sinensis*	HL60	*C. limon*: 0.625–2.5% v/v *C. sinensis*: 0.75–20% v/v	72 h	*C. limon:* IC50 1.4% *C. sinensis:* IC504.4%	Trypan blue exclusion method
Camarda et al., 2007	*C. aurantium* and *C. sinensi*s	K562 HL-60 MCF-7	10% v/v	48 h (K562, HL-60) 72 h (MCF-7)	39.7–100% (K562) 38.8–100% (HL60) 84.3–9.5% (MCF-7)	Trypan blue exclusion method (K562 and HL-60) MTT assay (MCF-7)
Dourado et al., 2015	*C. sinensis* red fleshed and blond	*Loucy*	*C. sinensis* juice at 10 μM of hesperidin	24 h	red fleshed: 23% blond: 27%	Trypan blue exclusion method
Celano et al., 2015	*C. reticulata* flavonoid rich extract	CAL-62 C-643 8505C	0.001–0.5 mg/ml	24 and 48 h	The strongest inhibitory effect was observed after 48 h of treatment with 0.5 mg/ml concentration. CAL-62: 70% C-643: 60% 8505C: 50% ^*^	MTT and cell count assays
Raimondo et al., 2015	*C. limon* nanovesicles	A549 SW480 LAMA84	5 or 20 μg/ml	24-72 h	20 μg/ml at 48 h: 50% all cells	MTT assay
Gharagozloo et al., 2002	*C. aurantifolia* concentrated extract	MDA-MB-453 RPMI 8866	0, 15-500 μg/ml	24 h	No significant effect on MDA-MB-453 but significantly inhibited spontaneous proliferation of RPMI 8866 125 μg/ml: 47.7% 250 μg/ml :46.2% 500 μg/ml :49.9%	Trypan blue exclusion method
Patil et al., 2009	*C. aurantifolia extracts*	PANC-28	6.25–200 μg/mL (MTT assay) 25–100 μg/mL (cell count assay)	24–72 h (MTT assay) 48–144 h (cell count assay)	24 h: IC50 > 200 μg/mL 48 h: IC50 109.67 ± 4.15- 121.82 ± 1.02 μg/mL 72 h: 81.20 ± 5.75- 101.60 ± 5.30 μg/mL ^*^	MTT and cell count assays
Entezari et al., 2009	*C. medica* half-ripe and ripe	Astrocytoma cells	25–500 μl/ml	48 h	Half ripe: 50–100% ripe: 70–100%	MTT assay
Delle Monache et al., 2013	*C. aurantium* spp. *bergamia*	PC3 MDA-MB231 PC12 SH-SY5Y	0.5–5% v/v	24–72 h	The juice showed antiproliferative activity in all tested cancer cell lines. The greatest inhibitory effect (64 ± 4%) was reported in SH-SY5Y after 72 h to 5% concentration ^*^	MTT and cell count assays
Visalli et al., 2014	*C. aurantium* spp. *bergamia flavonoid rich extract*	HT-29 Caco-2	0.1–5 mg/ml	24–72 h	24 h 2.5 mg/ml: 35% 5 mg/ml: 60% 48 and 72 h 5 mg/ml >80%^*^^#^	MTT and BrdU assays
Ferlazzo et al., 2016b	*C. aurantium* spp. *bergamia*	HepG2	0.5–10% v/v	24–72 h	The maximal antiproliferative effect (75%) was observed after 72 h exposure to 10% v/v concentration ^*^	MTT and cell count assays

* *These data refer to the MTT assay*.

# *These data refer to HT-29 cells*.

**Table 3:** Principal characteristics of the *in vivo* studies carried out using *Citrus* juices or their extracts.

**Study**	***Citrus* juices and extracts**	**Animal model**	**n° of animals**	**Sex**	**Cancer model**	**Dose of carcinogenic agent**	**Time of exposition to *C*. juices or extracts**	**Dose of *C*. juices or extracts**
So et al., 1996	*C. sinensis C. paradisi*	Sprague-Dawley rats	Five groups of 21 each	F	DNBA- induced mammary tumors	Single dose 5 mg of DMBA	15 weeks	*Ad libitum*
Miyagi et al., 2000	*C. sinensis*	Fischer 344 rats	Two groups of 30 each	M	AOM-induced colon cancer	Two doses of 15 mg/kg bw	28 weeks	*Ad libitum*
Tanaka et al., 2000	*C. sinensis* rich in beta-cryptoxanthin and hesperidin	Fischer 344 rats	113	M	AOM-induced colon cancer	2 weekly of 20 mg/kg bw	36 weeks	*Ad libitum* for 12 hr/day and tap water for the remaining 12 h.
Tanaka et al., 2012	*C. sinensis* rich in beta-cryptoxanthin and hesperidin	Fischer 344 rats	32	M	AOM-induced colon cancer	2 weekly of 20 mg/kg bw	4 weeks	500 ppm
Kohno et al., 2001	*C. sinensis*	A/J mice	103	M	NNK- induced pulmonary neoplasm	10 micromol in saline/mouse	21 weeks	*Ad libitum* in the night
Madrigal-Bujaidar et al., 2013	*C. paradisi*	CF-1 mice	Six groups of 16 each	F	AOM-induced colon aberrant crypt formation	10 mg/kg twice a week for 2 weeks	7 weeks	0.8, 4.1, and 8.2 μl/g
Miyata et al., 2002	*C. paradisi*	Fischer 344 rats	na	M	PhIP- induced colon DNA damage	60 mg/kg	5 days	*Ad libitum*
Navarra et al., 2014	*C. aurantium* spp. *bergamia*	SCID mice	48	M/F	SK-N-SH and LAN-1-induced pulmonary metastases	1 × 10^6^ cells	28 days	200 μl/day

**Table 4:** Main characteristics of observational studies.

**Study**	***Citrus* juices**	**Study design**	**Study population**	**Primary objective**	**Method of dietary assessment**	Measures^*^	**IC95%**
Maserejian et al., 2006	*C. sinensis*	Prospective cohort study	42,311 men aged 40–75 years when the study began	Evaluate fruit and vegetable consumption and the incidence of OPLs	Interview using 131-item FFQ	RR 0.65	0.42–0.99
Jansen et al., 2011	*C. sinensis* and *C. paradisi*	Clinic-based case-control study	2,473 cases 2,708 controls	Evaluate fruit, vegetable, fiber, and grain consumption associations with pancreatic adenocarcinoma	Interview using 144-item FFQ	OR 0.52	0.35–0.79

* *Comparing highest to lowest quintiles*.

### *Citrus* juices and their extracts in cancer: focus on preclinical studies

*Citrus* fruits (CF) originated from tropical and sub-tropical areas of South-East Asia and were imported into Europe during the times of the Roman Empire. In Mediterranean basin, CF found favorable climatic conditions leading to extensive cultivation and the origin of many new hybrids (Duarte et al., 2016). Hence, *Citrus* taxonomy is quite controversial, so that there are different attempts to classify this Rutaceae family (Swingle and Reece, 1967; Tanaka, 1977; Mabberley, 1997). According to recent taxonomy, there are three ancestral taxa: *Citrus maxima* (pomelos), *Citrus reticulata* (mandarins), and *Citrus medica* (citrons) (Garcia-Lor et al., 2013). CF exerts many beneficial effects that improve human health, including mainly anti-oxidant, anti-inflammatory, anti-microbial and anticancer effects, as well as protection against cardiovascular and nervous system injury (Lv et al., 2015; Mandalari et al., 2017). As recently stated by Cirmi and co-workers, an impressive number of preclinical studies explored the anticancer activity of *Citrus* flavonoids as single compounds (Cirmi et al., 2016b). Conversely, limited investigations tested the potential of *Citrus* juices or their extracts against cancer.

In this systematic review, we included and discussed twenty preclinical studies in which *Citrus* juices or their extracts were used as anticancer agents. In particular, twelve papers evaluated the effect of *Citrus* juices in *in vitro* experimental models, and eight in *in vivo* ones.

*Citrus sinensis* (orange) is a small tree originated in China and then imported to Europe between sixteenth and seventeenth century by Portuguese expeditions (Ferrão, 1979). It is described as a hybrid between *Citrus maxima* (pomelo) and *Citrus reticulata* (mandarin). Orange juice (OJ) is one of the most consumed among other CF juices worldwide, and even one of the most studied regarding its health benefits. Indeed, it is well known its antioxidant property that is due to its content of both Vitamin C and flavonoids (Constans et al., 2015). In this regard, recently we studied the antioxidant activity of an extract from orange juice (OJe) rich in flavonoids in both *cell-free* and *cell-based* experimental models. In particular, we showed its capability of preventing oxidative stress induced by hydrogen peroxide (H_2_O_2_) in human lung epithelial A549 cells, diminishing reactive oxygen species (ROS) generation and membrane lipid peroxidation, as well as enhancing mitochondrial functionality and averting DNA-oxidative damage (Ferlazzo et al., 2015). Moreover, we demonstrated the iron-chelating activity exerted by OJe in Fe_2_SO_4_-exposed A549 cells and its induction of the antioxidant catalase, thus stopping oxidative injury induced by iron (Ferlazzo et al., 2016c). We further studied the antioxidant and anti-inflammatory activity of OJe through an animal model resembling colitis (Fusco et al., 2017) in which it counteracted cytokine generation, nuclear factor kappa B (NF-kB) activation, nitrotyrosine and poly ADP-ribose (PAR) formation, as well as enhanced myeloperoxidase (MPO) activity, increased manganese-dependent superoxide dismutase (MnSOD) expression and modulated both pro- and anti-apoptotic factors. Finally, it has been demonstrated that both OJ and OJe are capable of reducing cardiovascular risks (Buscemi et al., 2012) and exert anti-epileptic effect (Citraro et al., 2016). We found seven preclinical studies on the anticancer activity of OJ matching the eligibility criteria of our review. Among these, four have been investigated in *in vitro* experimental models (Kawaii et al., 1999; Camarda et al., 2007; Fernandez-Bedmar et al., 2011; Dourado et al., 2015) and two in animal models (So et al., 1996; Miyagi et al., 2000). Through *in vitro* study, Kawaii and co-workers screened 34 *Citrus* juices for their antiproliferative activity on several cancer cell lines (A549, human lung carcinoma; B16 mouse melanoma 4A5; CCRF-HSB-2, T-cell leukemia; TGBC11TKB, human gastric adenocarcinoma) and non-cancer cell line (HUVEC, human umbilical vein endothelial cells; HFK normal human foreskin keratinocytes) (Kawaii et al., 1999). *Citrus* juices tested included *C. bergamia, C. limon, C. grandis, C. paradisi, C. aurantium, C. sinensis, C. nobilis, C. unshiu, C. reticulata, C. tangerine*, and *C. clementina*. Although with different strength, all the tested juices showed antiproliferative activity, with the most exerted by *C. nobilis* (Kawaii et al., 1999). The growth inhibitory effect of both lemon and orange juices was also assessed by Fernández-Bedmar and collaborators on human leukemia HL-60 cells, suggesting a stronger activity of lemon rather than orange juice (IC_50_ 1.4 and 4.4%, respectively) (Fernandez-Bedmar et al., 2011). Moreover, Camarda and co-workers showed the antiproliferative effect of OJ and some varieties of *C. reticulata* in human chronic myelogenous leukemia (CML) K562, human breast adenocarcinoma MCF-7 and HL-60 cells (Camarda et al., 2007). Finally, both sweet red-fleshed and blond OJ exert pro-apoptotic effect on *Loucy* T acute lymphoblastic leukemia cell line (Dourado et al., 2015). The first study assessing the inhibition of rats' mammary tumor generation caused by 7,12-dimethylbenz[a]anthracene (DMBA) through OJ treatment was performed in 1996 by So et al. (1996). Some years later, Miyagi and collaborators evaluated the capability of OJ to inhibit azoxymethane (AOM)-induced colon tumor in male rats, indicating that flavonoids and limonoid glucosides are implied in this antitumor activity (Miyagi et al., 2000).

*Citrus reticulata* (mandarin), originated from South-East China, was imported in Europe just in the nineteenth century, where it was cultivated mainly in Italy and Spain (Zaragoza, 1991), and represents the ancestor of different hybrids and mutants cultivated throughout the world (Scora, 1988; Mabberley, 2009). The main representative *Citrus reticulata* varieties in the Mediterranean area are *Citrus deliciosa, Citrus unshiu*, and *Citrus clementina* (Tanaka, 1954). Navarro and co-workers have studied the juice of mandarin (MJ) growing in Mediterranean area for its antioxidant activity (Navarro et al., 2011), while Oikeh et al. demonstrated its anti-microbial activity, in some cases, better than other CF (Oikeh et al., 2016). We analyzed five preclinical studies in which MJ was tested for its potential anticancer activity. In particular, we found two *in vitro* (Camarda et al., 2007; Celano et al., 2015) and three *in vivo* studies (Tanaka et al., 2000, 2012; Kohno et al., 2001). As mentioned above, OJ, *C. clementina* and *C. deliciosa* juices exert antiproliferative effect in K562 and HL-60 cells (Camarda et al., 2007). Lately, Celano and co-workers have examined the anticancer properties of a mandarin juice extract rich in flavonoids (MJe) on three diverse cell lines of human anaplastic thyroid carcinoma (CAL-62, 850C, and C-643). In this study, we demonstrated that MJe blocks cell cycle in G2/M phase, causing a reduction in cell proliferation and an increase in autophagic cell death (Celano et al., 2015). Furthermore, MJe reduced activity of MMP-2, thus decreasing cell migration (Celano et al., 2015). In animal models, Tanaka and collaborators showed the ability of natural MJ and MJs enriched in hesperidin and β-cryptoxanthin to reduce the AOM-induced colon carcinogenesis in F344 rats (Tanaka et al., 2000). Moreover, the juices cited above were also found to reduce lung neoplasms induced by 4-(methylnitrosamino)-1-(3-pyridyl)-1-butanone (NNK) in A/J mice (Kohno et al., 2001). Finally, these MJs, both natural and enriched, decreased the gene expression of tumor necrosis factor (TNF)-α, interleukin (IL)1-β, IL-6, NF-E2-related factor 2 (Nrf2), COX-2, and iNOS in the tongue and colon of F344 rats subjected to 4-Nitroquinoline-1-Oxide (4-NQO) or AOM to induce carcinogenesis, respectively (Tanaka et al., 2012).

*Citrus paradisi* (grapefruit) is thought to be a natural hybrid between *Citrus maxima* and *Citrus sinensis* that was found in the Caribbean after the discovery of the Americas by Columbus, and then exported to Europe (Barrett and Rhodes, 1976; de Moraes et al., 2007; Ollitrault et al., 2012). Grapefruit juice (GJ) has been extensively studied for its pharmacokinetic interactions with many drugs (Ahmed et al., 2015) and for its antioxidant and anti-inflammatory properties (de la Garza et al., 2015). Only two studies performed in animal models explored the anticancer properties exerted by GJ. Madrigal-Bujaidar and co-workers showed that GJ suppressed the colon carcinogenesis induced by AOM in mice, by up-regulating apoptosis and reducing both cyclooxygenase-2 (COX-2) and inducible nitric oxide synthases (iNOS) levels, thus suggesting even its anti-inflammatory activity (Madrigal-Bujaidar et al., 2013). Miyata and collaborators demonstrated that GJ also suppresses colon DNA-damage induced by 2-amino-1-methyl-6-phenylimidazo[4,5-b]pyridine (PhIP) in F344 male rats, however without identifying the mechanism of action (Miyata et al., 2002).

*Citrus limon* (lemon) has uncertain origin, even if it is thought to come from South-East Asia, as reported in data from Chinese literature of fifth century BC (Duarte et al., 2016). Then, Arabs brought this *Citrus* in Europe in twelfth century (Duarte et al., 2016). *Citrus limon* is known to be a hybrid between *Citrus medica, Citrus reticulata*, and *Citrus maxima* (Garcia-Lor et al., 2013). Lemon juice (LeJ) is thought to have, among others, anti-bacterial (Yang et al., 2013) and anti-oxidant activities (Tounsi et al., 2011). Only the paper by Raimondo et al. met the inclusion criteria of our review and its content is discussed below. Authors demonstrated that the nanovescicles extracted by ultracentrifugating LeJ might induce apoptosis in CML cells by activating TRAIL-mediated cell death (Raimondo et al., 2015).

*Citrus aurantifolia* (lime) is a tree originated in the tropical Northern Indian area. It was brought by Arabs into Middle East, Northern Africa and Mediterranean Europe, and then Spanish expeditions spread this *Citrus* in Central and South Americas. It is still widely cultivated in those areas cited above (Khan et al., 2017). *Citrus aurantifolia* is thought to be a hybrid between *C. medica* and *C. micrantha*, according to Nicolosi et al. (2000). Lime juice (LiJ) is claimed to exert antimicrobial activity on both Gram-positive and Gram-negative bacteria (Aibinu et al., 2006). Only two studies carried out *in vitro* tested the antiproliferative activity of LiJ (Gharagozloo et al., 2002; Patil et al., 2009). Ghargozloo and co-workers suggested that concentrated LiJ could reduce proliferation of human lymphoblastoid B cell line (RPMI-8866) (Gharagozloo et al., 2002). Furthermore, Patil and collaborators showed that LiJ extracted in different solvents including acetone, chloroform and methanol, modulates the expression of caspase-3, Bax, Bcl-2, and p53, thus inducing apoptosis in Panc-28 pancreatic cancer cell line (Patil et al., 2009).

*Citrus medica* (citron) is native of Indian region, and is known to be the first CF arrived in the Mediterranean basin, and both Greek and Latin literatures confirmed its presence as food in their diets. Indeed, its name came from “*Media*” which was the term that referred to Persian area that is where Greek discovered this fruit during Alexander the Great's expeditions (Duarte et al., 2016). As cited previously, *C. medica* is one of the three ancestral taxa from which every CF drew its origin (Garcia-Lor et al., 2013). We found only one *in vitro* study in which the antiproliferative activity of citron juice (CJ) was evaluated (Entezari et al., 2009). Authors demonstrated that CJ, at different ripeness stages, showed antiproliferative activity on 1321 human astrocytoma cancer cells. Moreover, they assessed the anti-mutagenicity activity of CJ through Ames test, in which the “half-ripe” CJ resulted stronger than the “ripe” one (Entezari et al., 2009).

*Citrus aurantium* ssp. *bergamia* (Risso and Poiteau) also called *Citrus bergamia* (Risso and Poiteau) or bergamot is thought to be a hybrid between *Citrus aurantium* L. and either *Citrus limon* L. (lemon) or *Citrus aurantiifolia* (lime). Additionally, it may be a mutation of the latter (Rapisarda and Germanò, 2013). It may originate from Canary Islands, Greece or Antilles and then introduced in Spain (“bergamot” can draw its name from the Spanish village “Berga”) and, subsequently, in southern Italy. More likely, it may be native of the Calabria region (Italy) where the best worldwide microclimate incentivizes both its plantation and spontaneous growth. Bergamot fruits are mainly used to extract the bergamot essential oil (BEO) mostly used in fragrance, cosmetics, confectionery and food industries. Moreover, BEO possesses antiseptic activities (Cirmi et al., 2016a) and it is used in aromatherapy (Navarra et al., 2015b). It is included in the official Pharmacopoeias of several countries, and its chemical composition has been accurately investigated (Costa et al., 2010). Experimentally, in the last decades, it has been investigated for its potential neuroprotective (Corasaniti et al., 2007) and antitumor activities (Celia et al., 2013; Navarra et al., 2015a). Bergamot juice (BJ), obtained through endocarp squeezing, has only been treated as a waste product of BEO factory chain for long time. Recently, it drew attention because of its hypolipemic and hypoglycaemic activity of its polyphenol fraction (Mollace et al., 2011; Toth et al., 2015; Mannucci et al., 2017). Furthermore, the flavonoid-rich extract from BJ (BJe) possesses both antioxidant and anti-inflammatory activities, as assessed in several *in vitro* (Risitano et al., 2014; Ferlazzo et al., 2015, 2016c; Curro et al., 2016) and *in vivo* models (Impellizzeri et al., 2015, 2016). In addition, it exerts antimicrobial effects (Filocamo et al., 2015; Mandalari et al., 2017). Evidence that BJ, BJe or other polyphenol extracts from BJ did not exhibit noticeably signs of systemic toxicity either in animals or in human suggests their valuable risk/benefit ratio (Marino et al., 2015). Four studies focused on the anticancer activity of BJ and BJe, in particular three performed *in vitro* (Delle Monache et al., 2013; Visalli et al., 2014; Ferlazzo et al., 2016b) and one *in vivo* (Navarra et al., 2014), and met the inclusion criteria of our systematic review. We have demonstrated that BJ is able to reduce growth rate in various cancer cell lines through diverse mechanisms. In neuroblastoma cells (SH-SY5Y) BJ caused cell cycle arrest in G1 phase without triggering apoptosis, and induced morphological change that in turn increased the rate of detached cells in different physiological substrates and in endothelial cell monolayer. This peculiarity was due to the ability of BJ to induce impairment of actin filaments and reduce expression of active form of focal adhesion kinase (FAK), thus inhibiting cell adhesiveness and migration (Delle Monache et al., 2013). Conversely, in human hepatocellular carcinoma cells (HepG-2) BJ diminished growth rate through the activation of both extrinsic and intrinsic apoptosis that involved NF-κB, p53, and p21 pathways (Ferlazzo et al., 2016b). Furthermore, the BJ-induced reduction of neuroblastoma cells (SK-N-SH and LAN-1) adhesiveness, as assessed *in vitro* through a laminar flow assay on selectins, might be responsible for the slight inhibition of pulmonary metastasis colonization in an *in vivo* model of neuroblastoma spontaneous metastases formation in SCID mice (Navarra et al., 2014). We further indicated that the pool of flavonoids presents in BJ is responsible for its anticancer effects, since BJe is able to inhibit the growth of HT-29 human colorectal carcinoma cells by induction of apoptosis that can be triggered through different mechanisms depending on the drug concentration (Visalli et al., 2014). Indeed, lower BJe concentrations suppressed MAPK pathways and modulated some proteins linked to the apoptotic machinery, thus inducing arrest of cell cycle and apoptosis. Instead, higher BJe concentrations raised ROS generation leading to loss of mitochondrial membrane potential (MMP) and DNA oxidative damage (Visalli et al., 2014).

### *Citrus* extracts in cancer: focus on observational clinical studies

In the last 25 years, a large body of evidence, mainly from epidemiological studies, suggests that regular intake of CF could prevent many diseases including cancer. Almost all the studies evaluating the anticancer potential of CF in humans come from case-control studies, but very few of them are performed using just the *Citrus* juices. We found only two studies that fall within the eligibility criteria of our systematic review. A very interesting prospective cohort study aimed to evaluate the impact of vegetables and fruits consumption and the oral premalignant lesions risk in 42,311 U.S. men was performed in the frame of the “Health Professionals Follow-up Study” (Maserejian et al., 2006). At baseline, participants aged 40–75 years completed detailed questionnaires assessing dietary intake, lifestyle factors, and medical history. Follow-up questionnaires were mailed every 2 years to update exposure information and ascertain newly diagnosed diseases. Authors observed a significant inverse associations with consumption of *Citrus* fruits and their juices (mainly orange and OJ) indicating 30–40% lower risk with greater intakes (e.g., *Citrus* fruit juice quintile 5 vs. quintile 1 RR = 0.65, IC 95%: 0.42, 0.99 (Maserejian et al., 2006). More recently, Jansen and co-workers used a clinic-based case-control study to demonstrate the inverse relationship between the consumption of high quantity of fibers, grain, fruits and vegetables and the risk of pancreatic cancer, suggesting that also orange and grapefruit juices might have a role in a pancreatic cancer prevention strategy (Jansen et al., 2011).

## Discussion

For more than a century now, people treated most diseases with synthetic drugs, which have defeated many illnesses and thereby allowed to increase the overall quality of life and its extension. In addition, over the last few decades the advent of biotechnological drugs has further improved the treatment of many chronic diseases. However, the best weapon to fight illnesses is always prevention. In particular, cancer prevention comes from healthy habits, as suggested by Tomasetti and co-workers who claimed that nearly 30–40% of cancer incidence might be prevented by a proper diet and physical activity in order to maintain appropriate body weight (Tomasetti and Vogelstein, 2015). However, sometimes it may be advisable to assume nutraceuticals, food supplements and natural remedies in order to improve one's natural defenses or to rebalance some loss of nutritional factors important to maintain the health status. On the contrary, employment of synthetic drugs for preventive purpose is not advisable, mainly because of the side effects that may arise. Indeed, even if natural products are not without risk, generally they are safer than synthetic drugs, have a reasonably effectiveness as well as meet patient's compliance. In this line, about 80% of people worldwide employs natural remedies to prevent and/or treat several illnesses. Since ancient times, edible plants and their fruits have been the bases of many traditional medicines, continuing to provide humankind with new remedies.

Many natural drugs are single compounds, some of these are implemented in clinical practice to prevent or treat several diseases, including cancer. Taxanes such as paclitaxel and Vinca alkaloids such as vinblastine are examples of active principles derived from plants that are prescribed in cancer therapy. However, our group (Delle Monache et al., 2013; Navarra et al., 2014; Visalli et al., 2014; Cirmi et al., 2016b; Ferlazzo et al., 2016b) and other researchers (Surh, 2003; Liu, 2004; Amin et al., 2009; Efferth and Koch, 2011) hypothesized that a single biologically active molecule, even if used at high concentrations, could not be sufficient in preventing or treating cancer because several different pathways are involved in malignant progression. Therefore, employment of complex mixtures of biologically active substances, such as those present in whole fruits and vegetables, juices or their extracts, increases the chances of success against cancer. The logic behind this hypothesis is: (i) the additive and synergistic actions of their individual components and (ii) their simultaneous modulation of different intracellular targets involved in oncogenesis. Thus, a cocktail of pharmacological actions is playing to induce the anticancer effect (Surh, 2003; Liu, 2004; Amin et al., 2009; Efferth and Koch, 2011; Delle Monache et al., 2013; Navarra et al., 2014; Visalli et al., 2014; Cirmi et al., 2016b; Ferlazzo et al., 2016b). The results of the present systematic review clearly indicate that *Citrus* juices and their extracts may exert antitumor effects, as suggested by the several studies discussed above that were performed in different *in vitro* and *in vivo* experimental models. These indications were strengthened by the data of observational clinical studies presented herein that showed how consumption of *Citrus* juices (in particular OJ and GJ) is inversely associated with the risk of oral and pancreatic cancer.

It is clearly known that cancer development and chronic inflammation are strictly intertwined each other and that inflammatory cells and released cytokines increase growth and progression of malignancies as well as immunosuppression (Balkwill and Mantovani, 2001). Furthermore, the imbalance of redox and inflammatory pathways between the inside of tumor cells and their surrounding stroma is known to be important in tumorigenesis, invasion and consequent systemic diffusion (Crawford, 2014). Additionally, pathways related to inflammation are constitutive in the majority of cancers. Consequently, the use of antioxidant and anti-inflammatory substances mainly derived from natural origins is highly desirable in the treatment of malignant tumors. Although these substances are not entirely risk-free, they are generally safer than synthetic drugs. Regarding this aspect, we recently showed that BJe possesses both antioxidant (Ferlazzo et al., 2015, 2016c) and anti-inflammatory activities, the latter through *in vitro* (Risitano et al., 2014; Curro et al., 2016) and *in vivo* studies (Impellizzeri et al., 2015, 2016). We also demonstrated the antioxidant and anti-inflammatory activity of OJe (Ferlazzo et al., 2015, 2016c), as Tarozzi and collaborators claimed for the edible part of orange (Tarozzi et al., 2006), as well as its anti-inflammatory effect (Fusco et al., 2017). These properties corroborate the anticancer activity of the *Citrus* juices and their extracts, rising their potential use. However, their chemopreventive activity should be tested in clinical trials in order to strengthen the value of preclinical data presented in this systematic review. Indeed, despite the large body of evidence of pre-clinical studies showing *Citrus* juices anticancer effect, a current limitation for its antitumor employment is that clinical studies are only a few and their results were obtained through self-assessed tests on patients.

## Conclusion

The present review has summarized the current status of the anticancer effects of *Citrus* juices and their extracts. Our study highlights the importance of *Citrus* juices and their extracts in a multitargeted-pharmacological strategy, suggesting their role in the prevention of cancer as well as their possible use as co-adjuvants in modern oncological therapies. However, further experimental and clinical studies are needed to exploit the beneficial aspects of these juices and their extracts in full.

## Author contributions

SC: performed the systematic literature searching, the data extraction and helped in drafting the paper; AM: carried out the data extraction and contributed in writing the paper; NF: participated in the creation of the paper; US, GC, and SG: critically revised the paper; MN: conceived and designed the study, performed the systematic research and the data extraction, as well as drafted the paper. All authors read and approved the final manuscript.

### Conflict of interest statement

The authors declare that the research was conducted in the absence of any commercial or financial relationships that could be construed as a potential conflict of interest.

## Footnotes

^1^(IARC) IAfRoC. (2014). World Cancer Report 2014.

## Abbreviations

4-NQO: 4-Nitroquinoline-1-Oxide
AOM: azoxymethane
Bax: bcl-2-like protein 4
Bcl-2: B-cell lymphoma 2
BEO: bergamot essential oil
BJ: bergamot juice
BJe: bergamot juice extract
CF: *Citrus* fruits
CJ: citron juice
COX-2: cyclooxygenase 2
DMBA: 7,12-Dimethylbenz[a]anthracene
FAK: focal adhesion kinase
H_2_O_2_: hydrogen peroxide
IL1-β: interleukin 1 beta
IL-6: interleukin 6
iNOS: inducible nitric oxide synthase
LiJ: lime juice
MAPK: mitogen-activated protein kinase
MJ: mandarin juice
MJe: mandarin juice extract
MMP: mitochondrial membrane potential
MMP-2: matrix metalloproteinase-2
MnSOD: manganese-dependent superoxide dismutase
MPO: myeloperoxidase
NF-kB: nuclear factor kappa B
NKK: 4-(methylnitrosamino)-1-(3-pyridyl)-1-butanone
Nrf2: NF-E2-related factor 2
OJ: orange juice
OJe: orange juice extract
PAR: poly ADP-ribose
PhIP: 2-amino-1-methyl-6-phenylimidazo[4,5-b]pyridine
PRISMA: Preferred Reporting Items for Systematic Reviews and Meta-Analyses
ROS: reactive oxygen species
SCID: severe combined immunodeficiency
TNF-α: tumor necrosis factor
TRAIL: TNF-related apoptosis-inducing ligand.
